# The Roles of Social Value Orientation and Anticipated Emotions in Intergroup Resource Allocation Decisions

**DOI:** 10.3389/fpsyg.2020.01455

**Published:** 2020-07-07

**Authors:** Suzanna Awang Bono, Job van der Schalk, Antony S. R. Manstead

**Affiliations:** ^1^School of Social Sciences, Universiti Sains Malaysia, Penang, Malaysia; ^2^School of Psychology, Cardiff University, Cardiff, United Kingdom

**Keywords:** social value orientation, anticipated emotions, ingroup favoritism, allocation behavior, economic games

## Abstract

How individuals divide resources between themselves and another person is influenced both by their social value orientation (SVO) and the emotions they expect to feel when dividing resources (anticipated emotions). Research has also shown that individuals favor members of their own group (ingroup) over individuals from other groups (outgroup) when allocating resources. The Malaysian multi-ethnic population is a highly relevant context to study the combined effects of intergroup relations and SVO on anticipated emotions and resource allocation. The current studies recruited Malaysian participants to examine whether anticipated emotions and allocation behavior are influenced by the ethnic identity of the person receiving the allocation. Participants completed an SVO measure and rated how they would feel if they were to share resources equally or unequally. They then made allocations between themselves and an ingroup or outgroup member in an economic game. There was no evidence of ingroup favoritism in anticipated emotions and allocation behavior. This may have been due to impression management, social desirability concerns, and/or the use of a population with socially liberal attitudes. The results nevertheless provide support for the notion that anticipated emotions play a key role in resource allocation decisions.

## Introduction

Both individual dispositional preferences and anticipated emotions are known to affect fairness and cooperative behavior (Mellers and McGraw, 2001; Van Der Schalk et al., 2012). For instance, allocators with a dispositional preference to be fair anticipate more cooperative emotions and fewer competitive emotions when allocating resources, and these anticipated emotions lead to fairer allocations to others (Bono et al., unpublished). At the same time, individuals tend to favor members of their own group (ingroup) over individuals from other groups (outgroup) when allocating resources (Ben-Ner et al., 2009). In the current research, we investigate whether the emotions that an individual anticipates when deciding how to allocate resources and the actual resource allocations are influenced by the social identity of the person receiving the allocation. We also explore whether this is moderated by individual differences in social value orientation (SVO).

Individual differences in SVO are known to affect the way that individuals allocate resources in an economic game (Messick and McClintock, 1968). SVO is commonly categorized into three orientations; prosocial, individualistic, and competitive (Van Lange et al., 1997). *Prosocials* prefer to minimize the difference in resources between themselves and others or to maximize both their own and others’ outcomes. *Individualists* prefer to maximize their own payoff. *Competitive* individuals prefer to maximize the difference between their own and others’ outcomes. In past research, individualists and competitors are usually combined into a single “proself” category (Haesevoets et al., 2015), a term that we will adopt here.

Individual differences in SVO have been demonstrated to affect behavior in experimental social dilemmas such as the prisoner’s dilemma, the public goods dilemma, and the commons dilemma (Balliet et al., 2009). Because prosocials tend to choose options that maximize joint outcomes, they cooperate more than proselfs do. Proselfs tend to choose outcomes that benefit themselves and therefore cooperate less. In a recent meta-analysis (Pletzer et al., 2018), prosocials and proselfs were found to differ in their expectations of others; more specifically, prosocials expect others to cooperate more, and this increases their cooperative behavior.

Other economic games that have been used to investigate the relation between SVO and allocation behavior include the ultimatum game (UG; Güth et al., 1982) and the dictator game (DG; Kahneman et al., 1986). Both games involve two players who have distinct roles. One (the allocator) makes an allocation between him/herself and the other (the receiver). The games differ only with respect to the role of the receiver. In the UG, the receiver can either accept or reject the proposed allocation. If the allocation is accepted, the allocator and receiver receive the proposed allocation. If the proposed allocation is rejected, neither player receives anything. The UG allocation therefore contains a strategic component. The allocator has to estimate the level at which his or her offer might be rejected by the receiver. In the DG, on the other hand, the receiver has to accept the proposed allocation made by the allocator. The DG is therefore considered to be a “purer” measure of fairness in allocation behavior. Because no strategic component is involved, it is assumed that allocators behave in accordance with their dispositional preferences.

Past research has also shown that individuals are affected by the emotions that they anticipate experiencing as a consequence of their decision making. For example, anticipated pride about being fair and anticipated regret about being unfair predicts cooperative resource allocation behavior (Van Der Schalk et al., 2012). Similarly, an individual may behave in a more desirable way (perhaps more morally) in order to avoid feeling disappointment (Zeelenberg et al., 2000; Gill and Prowse, 2012). In a study investigating divorce negotiation, guilt was reported to enhance cooperative behaviors (Wietzker et al., 2012). Feeling shameful when one fails to act morally was also found to motivate prosocial behavior (De Hooge et al., 2008). Furthermore, results show that anticipated emotions can both increase and decrease cooperative and competitive behaviors. For example, anticipating feeling proud about being unfair can lead people to share less of their resources (Van Der Schalk et al., 2012; see also Dorfman et al., 2014). Similarly, anticipated regret and guilt about being fair can lead people to share fewer resources (Ketelaar and Tung Au, 2003; Van Der Schalk et al., 2012).

Bono et al. (unpublished) investigated the relation between SVO, anticipated emotions, and allocation behavior, and experimentally varied anticipated emotions by means of an emotion regulation manipulation. Results showed that anticipated emotions function as a psychological link between individual differences in SVO and resource allocation decisions, and there was evidence suggesting that the typically observed relation between SVO and allocation behavior was disrupted when individuals were instructed to upregulate or downregulate their anticipated emotions. Overall, Bono et al. (unpublished) showed that allocators with a dispositional preference to be fair (prosocials) anticipated more cooperative emotions (pride about being fair, regret about being unfair), and that these cooperative emotions help to explain why prosocials tend to make more fair decisions.

Social identity theory (Tajfel and Turner, 1979) argues that individuals derive part of their identity from the groups they belong to and that this contributes to their self-esteem. Individuals are therefore motivated to find attributes of their groups that positively distinguish them from other groups. In an intergroup setting, social identity becomes salient and individuals are inclined to search for positive ingroup differentiation. Some researchers have used economic games to study differences in allocation behavior toward ingroup and outgroup members, and found that allocators gave more resources to ingroup members than to outgroup members (Ben-Ner et al., 2009; Balliet et al., 2014). Participants also tend to be more cooperative or prosocial toward others with whom they share a cultural/national background than to those with a dissimilar cultural/national background (Fiedler et al., 2018). This ingroup favoritism, or tendency to favor ingroup members over outgroup members, is consistent with the social identity theory argument that group members will search for ways to distinguish the ingroup from an outgroup in ways that reflect well on the ingroup. Furthermore, there is evidence that the extent to which group members identify with their ingroup moderates ingroup favoritism, such that high identifiers are more likely to display such favoritism (e.g., Hinkle et al., 1989; Sidanius et al., 1994; Levin and Sidanius, 1999).

In the current studies, we investigate how the multi-ethnic context of Malaysia affects the emotions that citizens expect to feel when allocating resources equally or unequally between themselves and others, and how they actually make such allocations. Malaysia’s population consists of three main ethnic groups, Malay (69.1%), Chinese (23%), and Indians (6.9%), and others (1%) (Department of Statistics, 2018). As a result of colonial history, Malays and other indigenous groups are labeled “*bumiputra*” (Siddique and Suryadinata, 1981; Khattab, 2016). “*Bumiputra*” means “*sons of the soil*” in the Malay language. This term is used to distinguish Malays and other indigenous groups from Chinese and Indians (*non-bumiputra*). *Bumiputras* have *bumiputra privileges*, which means they receive more educational and economic assistance from the Malaysian government (Pietsch and Clark, 2014). This policy was implemented by the Malaysian government in 1970 in its New Economic Policy (NEP) (Jomo and Sundaram, 2004; Brown, 2007) to help the *bumiputras* who, at the time, were faring less well than the *non-bumiputras*. This policy was also intended to help the different ethnic groups reach harmony, particularly in the economic and education field (Montesino, 2012; Mokhtar et al., 2017). However, this policy has raised issues of inequality between the *bumiputras* and *non-bumiputras* due to the amount of help the *bumiputras* receive from the government (Tyson et al., 2017). This in turn has also contributed to segregation between the ethnic groups within the society (Montesino, 2012; Cheong et al., 2016; Tyson et al., 2017). Because of the strained relations between the different ethnic groups, Malaysia is a relevant context in which to investigate differences in allocation toward ingroup and outgroup members. In the present research, we recruited participants from the three main ethnic groups: Malay, Chinese, and Indian. Each group is distinct in terms of culture, traditions, and religion. The Malays are the majority group (being the largest of the three) while Chinese and Indians are minority groups. When the terms “ingroup” and “outgroup” are used in the paper, this is always with reference to the ethnic group of the allocator. For example, for Malays, ingroup members are Malays and outgroup members are Chinese and Indians. Similarly, for Chinese participants, ingroup members are Chinese and outgroup members are Malays and Indians. The same applies to Indians, for whom outgroup members are Malays and Chinese. Bumiputra status is only relevant when it comes to dividing participants into members of “majority” versus “minority” groups. When these terms (“majority” and “minority”) are used, we divided the ethnic groups based solely on their “bumiputra” and “non-bumiputra” status, bearing Malaysian history in mind. Conducting the research in Malaysia also enabled us to examine whether Bono et al. (unpublished) findings would replicate in a non-Western culture, thereby addressing a known limitation of much psychological research (see Henrich et al., 2010).

The current research consists of two studies. In Study 1, our primary aim was to investigate differences in allocation behavior toward ingroup and outgroup receivers. We predicted that participants would allocate more tokens to ingroup members than to outgroup members. It is known that allocators are more likely to be cooperative (and therefore to make fairer allocations) toward ingroup than toward outgroup members. For example, previous research has shown that participants tend to be more cooperative or prosocial toward others with whom they share a cultural/national background than to those with a dissimilar cultural/national background (e.g., Fiedler et al., 2018). Given that individuals tend to prefer more equal outcomes between themselves and a member of their own group than between themselves and a member of an outgroup (Ben-Ner et al., 2009; Balliet et al., 2014), and given that individuals who are more likely to be fair in resource allocations tend to anticipate more cooperative emotions (e.g., Van Der Schalk et al., 2012), we hypothesize that individuals will experience more cooperative emotions when allocating to ingroup receivers then when allocating to outgroup receivers. We also predicted that ingroup identification would be positively associated with ingroup favoritism, given the previously mentioned evidence that high identifiers are more likely to engage in ingroup favoritism. A further aim was to examine how allocation behavior was affected by SVO. We examined whether in-group favoritism in allocation behavior would be moderated by SVO. We also investigated whether the effect of SVO on allocation behavior was mediated by anticipated emotions (Bono et al., unpublished). We predicted that prosocial participants would make larger allocations to others than would proself participants and would also anticipate more cooperative emotions, with the differences in allocation behavior being mediated by differences in anticipated emotions. In Study 2, our main aim was to re-examine the prediction that there would be a difference in allocation behavior toward ingroup and outgroup members. We also took the opportunity to explore the social dominance theory explanation for any differences in the allocation behavior of minority and majority group allocators toward others (regardless of the receiver’s ethnicity).

## Study 1

### Method

#### Design and Participants

The study had a 3 (Allocator group: Chinese, Indian, and Malay; quasi-experimental between-subjects factor) × 3 (Receiver group: Chinese, Indian and Malay; within-subjects factor) mixed design. We recruited 123 Malaysians (97 females, 25 males, one undisclosed, *M*_*age*_ = 25.23, *SD* = 2.94) from the three major ethnic groups, Chinese (*N* = 43), Indians (*N* = 38), and Malays (*N* = 42). Participants were 44 students, one unemployed, 78 employed. Recruitment was done through social media and snowballing. Each participant was given a RM15 (approximately £3) gift voucher for their time and were entered into a lucky draw in which four pairs had a chance to win a voucher worth RM60 (approximately £11) each. The questionnaire was administered online using a survey site (Qualtrics).

#### Materials

##### Index of cooperative and competitive emotion (ICE) measure

To measure anticipated cooperative and competitive emotions, participants were asked to rate how they would feel about division of tokens, using a scale of 1 (not at all) to 5 (very much) to indicate the extent to which they would feel each of six emotions: pleased, proud, regretful, disappointed, guilty, and ashamed. These six emotion terms were chosen to capture three emotion constructs: pride, regret, and guilt. The terms *pleased* and *proud* were selected to index pride; the terms *regretful* and *disappointed* were selected to index regret; and the terms *guilty* and *ashamed* were selected to index guilt. This was based on the shared positive valence of the terms pleased and proud (Tracy and Robins, 2007; Van Osch et al., 2018); the shared counterfactual character of regretful and disappointed (where the person experiencing the emotion can imagine a better state of affairs if he or she had acted or chosen differently; Zeelenberg and Pieters, 2007); and the shared self-blame character of the terms guilty and ashamed (where the person appears to feel that he or she is responsible for bringing about an unwanted state of affairs; Niedenthal et al., 1994; Haidt, 2003). The measure consists of 12 scenarios that represented equal [12:12 and 21:21 (Chinese), 9:9 and 24:24 (Indian), 15:15 and 18:18 (Malay)] and unequal [16:8 and 28:14 (Chinese), 12:6 and 32:16 (Indian), 20:10 and 24:12 (Malay)] allocations toward others who belonged to one of the three ethnic groups (Chinese, Indian, and Malay). For each ethnic group, ethnically appropriate names were used to designate the receiver, who was always the same gender as the allocator [Chinese: Siew Ling or Sui Mei (female) and Chi Yung or Jian Hong (male), Indian: Shantini or Lakshimi (female) and Viknesh or Kumar (male), and Malay: Nurul or Aini (female) and Ali or Samad (male)]. Each item was presented in random order. For example, one item asked participants to imagine that there were 36 tokens at stake, and the participant decides to allocate 24 tokens for him/herself and 12 tokens to the other person. Participants are then asked to rate how they would feel about this division of tokens. Definitions of each emotion were given in both English and the Malay language to make sure participants understood their meaning. English definitions were taken from the Oxford online dictionary and Malay definitions were taken from the Dewan Pustaka and Bahasa online dictionary.

For the anticipated emotions measure scores, we created index scores for cooperative and competitive emotions (ICE scores) by calculating the difference between responses on items relating to the fair and the unfair scenarios, in such a way that higher scores always reflected more cooperative emotions. For example, to calculate ICE-pride, the score for anticipated pride about being unfair was subtracted from the score for anticipated pride about being fair. Similarly, to calculate ICE-regret and ICE-guilt, the scores relating to fair scenarios were subtracted from the corresponding scores relating to unfair scenarios. These indices can be interpreted in such a way that a negative score reflects anticipating more competitive emotions, whereas a positive score reflects anticipating more cooperative emotions, with a zero indicating no difference in the anticipation of cooperative and competitive emotions. The correlations between the pride, regret, and guilt sub-scales of the measure were substantial (*r*s ranged from 0.721 to 0.925). For reasons of conciseness, below we report the results for the overall index of cooperative and competitive emotions, averaged across all emotion items (ICE). Cronbach’s alphas for the measure were high: ICE_Malay_ = 0.918, ICE_Chinese_ = 0.919, ICE_Indian_ = 0.915 (distinguished by receiver ethnic group, not respondent ethnic group).

##### Social value orientation

Social value orientation was assessed using the SVO Slider Measure (SVO-SM) (Murphy et al., 2011). This requires participants to choose their preferred allocation between themselves and the recipient (an anonymous other). The SVO-SM has six primary items. Each consists of nine allocation options, where each option corresponds to a certain amount of points to the allocator and to the receiver. Responses are used to calculate the degree of prosociality.

##### Allocation behavior

Each participant took the role of allocator in a DG (Kahneman et al., 1986). In this game, allocators decide how to divide resources between themselves and another, who has to accept the allocation. Allocators were given 30 tokens to divide between him/herself and an opponent who (by virtue of the same names being presented as in the ICE measure) belonged to one of the three ethnic groups. Participants were told that the tokens had monetary value, in the sense that tokens gained would be paid out in real money if they won a lottery. On completing the survey, participants were automatically entered into lucky draw in which they could win a gift voucher worth up to a maximum of RM60 (approximately £11).

##### Ingroup identity measure (IIM)

The IIM (Leach et al., 2008) assesses ingroup identification. This 14-item measure consists of two second-order factors: self-definition (which in turn consists of individual self-stereotyping and in-group homogeneity) and self-investment (which in turn consists of satisfaction, solidarity, and centrality). Example items are “I feel a bond with [ethnic ingroup]” (indexes solidarity) and “I have a lot in common with the average [ethnic ingroup]” (indexes individual self-stereotyping). Respondents were asked to rate the extent to which they agreed with each item on a scale ranging from 1 (strongly agree) to 5 (strongly disagree). The content of the IIM was tailored to each respondent’s ethnicity, such that respondents were only presented with items relating to their own ethnic ingroup. Cronbach’s alphas for the IIM were high: IIM_Malay_ = 0.909, IIM_Chinese_ = 0.919, and IIM_Indian_ = 0.953.

##### Attention check

An attention check presented participants with some text related to emotions and they were given three options to choose from. However, they were asked not to click on any of the options given and to move on to the next question. Participants failed the attention check if they clicked on one of the provided options.

#### Procedure

Participants first completed a consent form. Next, they completed demographic items (ethnic group, age, gender, fluency in English, and occupation). They were then asked to complete the SVO-SM and the IIM in a randomized order. Next, participants were shown the definitions of the emotions that they would be presented in the ICE measure. They then reported their anticipated emotions for the 12 different allocation scenarios of the ICE measure, in a randomized order. Next, participants completed the attention check and then played three DGs (one for each ethnicity: Chinese, Indian, and Malay, in a randomized order) to measure their allocation behavior. Participants were then asked whether they had taken their participation seriously. Finally, they were debriefed. Two pairs of participants were randomly picked for the lottery. They were paid out RM60 (approximately £11) that was the maximum possible winnings of the allocation in gift vouchers because participants were not actually paired with another participant.

### Results

#### Data Treatment

Of the 123 Malaysians recruited, data from 105 individuals (*M*_*age*_ = 25.33, *SD* = 2.86) were retained for analysis. There were 22 males, 82 females, and one participant with undisclosed gender. The retained participants included 35 Chinese, 33 Indians, and 37 Malays, of whom 34 were students, one was unemployed, and 68 were employed (two missing occupation data). Data from participants who failed the attention check (*N* = 7), admitted that they were not serious in answering the questionnaire (*N* = 3), or took longer than 2.5 times the median response time (*Mdn* = 19.35, *N* = 8) were dropped.

Exploration of the allocation data revealed that it was not continuous and not unbounded: The modal response for number of tokens offered was 15 and all other responses tended to be restricted to certain categories (e.g., 10). In light of this, we used non-parametric tests to investigate the effects of conditions on allocation behavior. For regression-based analyses (i.e., mediation analyses), we followed the procedure of Van der Schalk et al. (2015) by dichotomizing the allocation scores: offers ≥ 15 were recoded as “fair” and offers ≤14 were recoded as “unfair.” Regression-based analyses of the allocation behavior data were therefore calculated using logistic regression. For analyses of all other measures, we used parametric tests (i.e., *t*-test and ANOVA). Correlations between the measures can be found in Table 1.

**TABLE 1:** Means, SDs, and correlations for all key variables in Study 1.

	**Tokens allocated**	**Tokens allocated to ingroup receivers**	**Tokens allocated to outgroup receivers**	**SVO**	**ICE**	**IIM**
Tokens allocated to ingroup receivers	0.838^∗∗∗^	−	−	−	−	–
Tokens allocated to outgroup receivers	0.805^∗∗∗^	0.562^∗∗∗^	−	−	−	–
SVO	0.309^∗∗∗^	0.229^∗^	0.233^∗^	−	−	–
ICE	0.303^∗∗^	0.254^∗∗^	0.199^∗^	0.345^∗∗∗^	−	–
IIM	–0.132	–0.036	–0.132	–0.180	–0.333^∗∗∗^	–
*n*	103	103	103	105	104	104
*M*	13.83	13.79	13.86	30.13	1.17	4.58
*SD*	2.96	3.44	2.99	11.86	1.28	1.08

*^∗^*p* < 0.05, ^∗∗^*p* ≤ 0.01, ^∗∗∗^*p* ≤ 0.001.*

A sensitivity test using G^∗^Power software (Faul et al., 2007, 2009) showed that the current study had sufficient power (1 – β = 0.80) to find a small effect of Cohen *dz* = 0.025 (Cohen, 1969, p. 38).

#### Anticipated Emotions Toward Ingroup and Outgroup Receivers

A *t*-test showed that participants did not differ in their anticipated cooperative and competitive emotions toward ingroup (*M* = 1.14, *SD* = 1.32) and outgroup (*M* = 1.18, *SD* = 1.31) members, *t*(102) = -0.72, *p* = 0.471. It was further explored whether participants from each ethnic group differed in their anticipated cooperative and competitive emotions toward receivers from different ethnic groups by conducting a 3 (Allocator group: Chinese, Indian, and Malay; quasi-experimental between-subjects factor) × 3 (Receiver group: Chinese, Indian, and Malay; within-subjects factor) mixed ANOVA. There were no significant main effects for receivers’ ethnic group, *F*(2,202) = 2.14, *p* = 0.121, or for allocators’ ethnic group, *F*(2,101) = 1.82, *p* = 0.168 on anticipated emotions. There was also no significant interaction between receivers’ and allocators’ ethnic group, *F*(4,202) = 0.59, *p* = 0.672. This shows that Chinese, Indians, and Malays did not differ in their anticipated cooperative and competitive emotions toward the three ethnic groups.

#### Allocation Behavior

When comparing participants’ allocation behavior toward ingroup and outgroup receivers, a Wilcoxon signed rank test showed that participants did not differ in their allocation behavior toward ingroup (*M* = 13.79, *SD* = 3.44, *Mdn* = 15.00) and outgroup receivers (*M* = 13.86, *SD* = 2.99, *Mdn* = 15.00), *Z* = -0.24, *p* = 0.810. We further explored how each of the ethnic groups allocated their resources to the three receiver groups. We conducted three separate Friedman’s ANOVA tests, one for each allocator group, where we examined the allocations to three receiver groups. It was found that none of the ethnic groups differed significantly in their allocations to the ethnic receiver groups, χ*^2^_*Malay*_*(2) = 0.06, *p* = 0.972; χ*^2^_*Chinese*_*(2) = 1.40, *p* = 0.497; and χ*^2^_*Indian*_*(2) = 2.38, *p* = 0.304.

The Spearman’s correlations between SVO and allocations to ingroup and outgroup members were virtually identical (see Table 1), suggesting that although SVO was significantly associated with allocations to receivers in the expected fashion, the strength of this relation was not moderated by whether the receiver is an ingroup or outgroup member. This was confirmed by splitting the sample at the median SVO score into participants low and high in SVO, and using a Wilcoxon signed rank test to compare their allocations to ingroup and outgroup receivers. Participants low in SVO did not differ in their allocations to ingroup (*M* = 12.98, *SD* = 4.50, *Mdn* = 15.00) and outgroup receivers (*M* = 13.16, *SD* = 3.72, *Mdn* = 15.00), *Z* = -0.07, *p* = 0.944. Likewise, participants high in SVO did not differ in their allocation to ingroup (*M* = 14.64, *SD* = 1.27, *Mdn* = 15.00) and outgroup receivers (*M* = 14.61, *SD* = 1.67, *Mdn* = 15.00), *Z* = -0.41, *p* = 0.686. This again shows that the amount allocated to ingroup and outgroup receivers was not moderated by SVO.

To examine whether allocation behavior was affected by strength of ingroup identification, we calculated Spearman’s rho correlations between the IIM scores for each ethnic group and allocation behavior toward ingroup and outgroup members. None of the correlations between IIM scores and allocation behavior toward ingroup [*r*_*Malay*_(37) = -0.021, *p* = 0.903; *r*_*Chinese*_(35) = -0.089, *p* = 0.613; *r*_*Indian*_(31) = 0.001, *p* = 0.995] and outgroup members [*r*_*Malay*_(37) = -0.016, *p* = 0.927; *r*_*Chinese*_(35) = -0.219, *p* = 0.206; *r*_*Indian*_(31) = -0.152, *p* = 0.415] were significant.

For exploratory purposes, we also examined whether the ethnic groups differed in their overall allocation behavior. A Kruskal–Wallis test comparing Malay (*M* = 13.31, *SD* = 3.02, *Mdn* = 15.00), Chinese (*M* = 13.75, *SD* = 3.54, *Mdn* = 15.00), and Indian (*M* = 14.51, *SD* = 1.95, *Mdn* = 15.00) allocators showed that their allocation behavior toward others (irrespective of the receivers’ ethnicity) differed marginally from what would be expected by chance, *χ*^2^(2) = 5.38, *p* = 0.068. When participants were grouped according to their majority (Malays) and minority (Chinese and Indians) group status in the Malaysian society, a Mann–Whitney *U*-test showed that majority group allocators (*M* = 13.31, *SD* = 3.02, *Mdn* = 15.00) allocated significantly less to others (irrespective of the receivers’ ethnicity) than minority group allocators (*M* = 14.11, *SD* = 2.90, *Mdn* = 15.00), *U* = 947.50, *Z* = -2.317, *p* = 0.020.

We then explored the difference between the majority group and the minority group in anticipated cooperative and competitive emotions toward ingroup and outgroup members, using a 2 (majority vs. minority allocator, between-subjects) × 2 (ingroup vs. outgroup receiver, within-subjects) mixed ANOVA. There was no significant main effect of receivers’ group membership, *F*(1,101) = 1.01, *p* = 0.318, but there was a marginal main effect of allocators’ group membership, *F*(1,101) = 3.91, *p* = 0.051, on anticipated emotions toward others, whereby minority group participants anticipated more cooperative emotions (*M* = 1.34, *SD* = 1.34) than did participants belonging to the majority group (*M* = 0.85, *SD* = 1.13). The interaction between allocators’ and receivers’ group membership was not significant, *F*(1,101) = 1.20, *p* = 0.276.

#### Mediation Analyses

**FIGURE 1:**
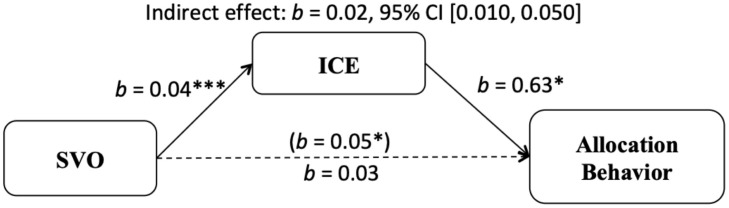
Indirect effect of social value orientation on dichotomized averaged tokens allocated to the receiver (irrespective of ethnicity) in the Dictator Game through ICE. *p < 0.05, ***p < 0.001 (Study 1).

Bono et al. (unpublished) found that the effect of SVO on allocation behavior was mediated by anticipated emotions. To investigate whether this pattern would be replicated in a non-Western sample, a mediation analysis using the PROCESS macro (Model 4) was carried out to test whether anticipated emotions (ICE) mediated the relation between SVO and allocation behavior (see Figure 1). This analysis showed that the total effect of SVO on tokens allocated in DG was positive and significant, and that there was a positive and significant effect of SVO on ICE, revealing that prosocials tended to allocate more to others and also anticipated more cooperative emotions. There was also a significant effect of ICE on allocation behavior. Moreover, the indirect effect of SVO on allocation behavior through ICE was significant, *b* = 0.02, 95% CI [0.010, 0.050], and the effect of SVO on allocation behavior was no longer significant when controlling for anticipated emotions, suggesting full mediation.

**FIGURE 2:**
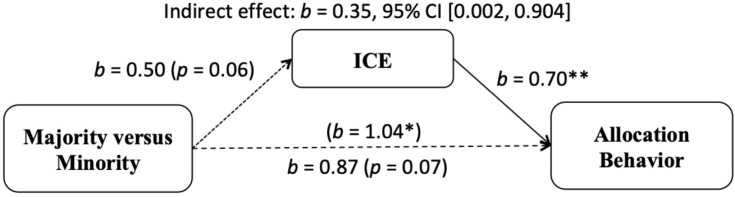
Indirect effect of allocator’s group membership (majority vs. minority) on dichotomized averaged tokens allocated to the receiver (irrespective of ethnicity) in the Dictator Game through ICE. *p < 0.05, **p < 0.01 (Study 1).

Because there were no significant effects of receivers’ group membership on allocation behavior or on allocators’ anticipated emotions, the indirect effect of group membership on allocation behavior via anticipated emotions could not be tested. However, because there was a significant difference in allocation behavior between the majority and minority groups, a second mediation analysis was conducted to test whether the effect of allocators’ group majority/minority status on allocation behavior was mediated by anticipated emotions (see Figure 2). This showed that the direct effect of group membership on tokens allocated was significant, and that the effect of group membership on ICE was marginally significant. In addition, ICE predicted allocation behavior, and the effect of group membership on allocation behavior was no longer significant when controlling for ICE. More importantly, the indirect effect of group on allocation behavior through ICE was significant, *b* = 0.35, 95% CI [0.002, 0.904].

### Discussion

The finding that differences in anticipated emotions mediated the effect of SVO on allocation behavior replicates the finding of Bono et al. (unpublished) that those with a prosocial disposition anticipate more cooperative emotions (pride about being fair, regret, and guilt about being unfair) and fewer competitive emotions (pride about being unfair, regret, and guilt about being fair), and that it is these anticipated cooperative and competitive emotions that are responsible for individual differences in allocation behavior. The current findings show that this pattern of mediation can be replicated in a non-Western culture.

Surprisingly, there was no support for the prediction that individuals would allocate fewer resources to outgroup members and would anticipate less cooperative emotions toward outgroup others. This stands in contrast to the ingroup favoritism in allocation behavior observed by other researchers (Forsythe et al., 1994; Berg et al., 1995; Ben-Ner et al., 2009; Liebe and Tutic, 2010). This may be because participants played three DGs consecutively with members of the three different ethnic groups, in a within-subjects design. This may have made them aware of the fact that the ethnicity of the other to whom they were making allocations was being varied. Impression management concerns may have led participants to refrain from allocating resources unequally between themselves and members of the different ethnic groups.

Nevertheless, when participants were re-classified into majority or minority groups, we found that minority group members were more likely than their majority group counterparts to make fair allocations to receivers and tended to anticipate fewer cooperative emotions (regardless of the receiver’s ethnicity). Social dominance theory (Pratto et al., 1994) offers a way to account for this difference in allocation behavior between majority and minority groups. This theory postulates that forming group-based hierarchies is a universal human tendency and that hierarchical social order is maintained through individual and institutional discrimination. In order for high status groups to maintain their position, they promote practices that enhance inequality. Lower status groups strive to be on par with the higher status group. The theory also identifies an individual difference in preference for hierarchical relationships between groups, which is termed social dominance orientation (SDO; Pratto et al., 1994). As noted earlier, in the Malaysian context, the majority (*bumiputras*) group enjoys higher status than the two minority groups (Chinese and Indians). In light of social dominance theory, it could be argued that the *bumiputras* acted less fairly in the present study in order to maintain prevailing status differences, whereas *non-bumiputras* acted more fairly in order to promote equality between groups. Research has also shown that advantaged group members have higher SDO scores than disadvantaged members, and that this is related to increased prejudice against disadvantaged members (Guimond et al., 2003). Likewise, it has been found that individuals higher in SDO are more likely to endorse social inequality and more likely to discriminate against members of disadvantaged groups (Ho et al., 2015).

Because this effect of majority/minority status on allocation behavior was found in an exploratory analysis, we sought to replicate the effect in a follow-up study. We also took the opportunity to measure respondents’ SDO. If differences in allocation behavior between majority and minority groups reflect differences in SDO, there should be a negative relationship between SDO scores and allocation behavior, such that individuals with a high SDO score, who prefer to maintain or even increase the differences in social status of different groups, should allocate less to others, perhaps especially when those others are members of lower status (minority) groups.

Additionally, despite the fact that the effect of group membership on ICE was only marginally significant, the significant indirect effect of group membership on allocations via anticipated cooperative emotions suggests that the difference in allocation behavior between majority and minority groups is, at least in part, explained by differences in anticipated emotions. This again illustrates the key role that anticipated emotions play in resource allocation decisions.

In Study 2, we changed the design of the study from a within-subjects to a between-subjects design, in order to minimize the influence of social desirability factors. By switching to a between-subjects design, the manipulation of the opponent’s social group identity should be less transparent. We also sought to recruit a bigger sample in order to rule out the possibility that the lack of evidence for differences in allocations to ingroup and outgroup members in Study 1 was due to lack of power. A further change from Study 1 was that we used the UG (Güth et al., 1982) instead of the DG. The UG differs from the DG in that the receiver has the option to reject a proposed allocation, in which case neither player receives any allocation. The UG therefore has a strategic component in the sense that the allocator needs to consider how the receiver would react to a proposed allocation, and this should increase participants’ engagement with the game.

## Study 2

The main aim of Study 2 was to re-examine the prediction that there would be a difference in allocation behavior toward ingroup and outgroup members. A further aim was to explore the social dominance theory explanation for the difference in allocation behavior of minority and majority group allocators toward others (regardless of the receivers’ ethnicity) found in Study 1. Social dominance theory argues that persons high in SDO have a preference for hierarchical social relations and are more accepting of inequality. Individual differences in SDO might therefore predict allocation behavior. A measure of SDO was added to investigate the extent to which preferences for group-based hierarchies could account for the effect of majority–minority groups status. We predicted that majority group members would have higher SDO scores than their minority group counterparts. We also explored the combined effects of allocator’s group membership (majority vs. minority), receiver’s group membership (ingroup vs. outgroup), and SDO on allocation behavior.

### Method

#### Design and Participants

This study had a 3 (Allocator group: Malay, Chinese, and Indian) × 3 (Receiver group: Chinese, Indian, and Malay) between-subjects design. There were 565 participants (435 females, 129 males, one other, *M*_*age*_ = 23, *SD* = 4.142) recruited for this study. Of these, 243 were Chinese, 222 were Malay, 65 were Indians, 22 were of mixed ethnicity, and 13 were from other ethnic groups. Participants were recruited from Malaysian universities through social media and mass emailing to groups of classes with the help of staff. Participants were all students. As an incentive, all participants were entered into a lucky draw in which four pairs had a chance to win a voucher worth RM60 (approximately £11) each. The questionnaire was administered through Qualtrics.

#### Materials

Materials were identical to those used in Study 1, with the following exceptions. The DG was replaced by the UG. SDO was measured using the scale developed by Pratto et al. (1994). The ICE measure of anticipated emotions was simplified by not varying the recipient’s ethnic identity, and the number of items was reduced from 12 to 6. When participants were asked how they would feel about making different types of allocation, they were reminded that the rules of the UG meant that the receiver could reject the allocation. An overall index of ICE was calculated using the same procedure as in Study 1. Once again, the three sub-scales (i.e., pride, regret, guilt) were found to be highly intercorrelated (with bivariate *r*s ranging from 0.611 to 0.891) and Cronbach’s alpha for the overall ICE measure was 0.927. The Cronbach’s alphas for the IIM were also high: IIM_Malay_ = 0.930, IIM_Chinese_ = 0.918, and IIM_Indian_ = 0.940, while that for the SDO measure was 0.837.

#### Procedure

A professional translator translated the questionnaire from English to Malay and the entire questionnaire was presented in both Malay and English. Participants were first asked to complete a consent form. They were then asked to provide demographic information (ethnicity, age, gender, fluency in English and Malay, and occupation). Next, participants completed the IIM, then the SVO-SM and the attention check. This was followed by the SDO measure and the ICE measure. Next, participants played the UG once with an opponent whose name was randomly chosen from the three ethnic groups. Each participant played the role of the allocator and was given a total of 30 tokens, to be divided between him/herself and the receiver. The names used were the same as in Study 1 and the assigned receiver was always the same gender as the allocator. Participants were told that the receiver would be able to accept or reject the proposed allocation, and that if the recipient rejected the proposal, neither the allocator nor the recipient would receive any tokens. On the other hand, if the recipient accepted the proposal, the allocator and the recipient would receive what the allocator had proposed. Participants were told that the tokens had monetary value in the sense that the points gained would be doubled and would be paid out in real money if they won the lucky draw. Participants were then asked whether they had taken their participation in the study seriously. Finally, participants were thanked and debriefed. In order to divide the gift voucher according to participants’ allocation in the UG, we needed to retrieve the minimal offer that each participant would accept. However, this information was not collected in the study. Because of this, each winner was given the maximum amount that they could win which was RM60 (approximately £11) in gift vouchers.

### Results

#### Data Treatment

Of the 565 participants, data from 371 participants (*M*_*age*_ = 23.05, *SD* = 4.06) were retained for analysis. There were 81 males and 290 females in the final sample. We excluded data from participants who failed the attention check (*N* = 62) and whose response time was either shorter than 2.5 times the median response time (*N* = 37) or longer than 2.5 times the median response time (*N* = 29). We also excluded participants whose ethnicity was “other” (*N* = 1) or mixed (*N* = 16). Due to the low number of ethnic Indian participants recruited, data from these participants (*N* = 49; *n*_*ChineseReceivers*_ = 18, *n*_*IndianReceivers*_ = 15, *n*_*MalayReceivers*_ = 16) were also not included in our main analyses. Thus, Chinese (*N* = 197) and Malay (*N* = 174) allocators were included in the data analyses.

As in Study 1, we used non-parametric tests to investigate the effect of condition on allocation behavior. For the regression-based analyses (e.g., mediation analysis), we dichotomized the allocation scores and calculated effects of predictors with logistic regression, with offers ≥ 15 coded as “fair” and offers ≤ 14 coded as “unfair.” For all other analyses, *t*-tests were used. See Table 2 for the correlations between the measures.

**TABLE 2:** Means, SDs, and correlations for all key variables in Study 2.

	**Tokens allocated**	**SVO**	**ICE**	**IIM**	**SDO**
SVO	0.283^∗∗∗^	–	–	–	–
ICE	0.301^∗∗∗^	0.329^∗∗∗^	–	–	–
IIM	−0.143^∗∗^	−0.124^∗^	–0.093	–	–
SDO	−0.158^∗∗^	−0.124^∗^	–0.325^∗∗∗^	0.042	–
*M*	14.48	31.38	1.00	4.96	43.12
*SD*	2.88	10.77	1.18	0.96	11.82

*Note: *N* for all variables = 370 except for SDO, where *n* = 369. ^∗^*p* < 0.05, ^∗∗^*p* ≤ 0.01, ^∗∗∗^*p* ≤ 0.001.*

A sensitivity test (using G^∗^Power) showed that the study had sufficient power (1 – β = 0.80) to find a small effect of Cohen dz = 0.026 (Cohen, 1969, p. 38).

#### Allocation Behavior

A Mann–Whitney test was used to investigate whether participants differed in allocations to ingroup and outgroup members. Allocations to ingroup members (*M* = 14.53, *SD* = 2.96, *Mdn* = 15.00) did not differ significantly from allocations to outgroup members (*M* = 14.46, *SD* = 2.84, *Mdn* = 15.00), *U* = 15414.50, *Z* = −0.19, *p* = 0.852. Using a Kruskal–Wallis test, we found that neither ethnic group (Malay or Chinese) differed significantly in its allocation behavior toward receivers from the different ethnic groups, χ*^2^_*Malay*_*(2) = 0.42, *p* = 0.813 and χ*^2^_*Chinese*_*(2) = 0.51, *p* = 0.775.

Next, we compared the allocation behavior of the majority and minority group members toward others (irrespective of ethnicity) using a Mann–Whitney test. Results showed that majority (*M* = 14.38, *SD* = 3.07, *Mdn* = 15.00) and minority (*M* = 14.57, *SD* = 2.71, *Mdn* = 15.00) groups did not differ in allocations, *U* = 16490.50, *Z* = -0.76, *p* = 0.450. This is inconsistent with what was found in Study 1. We also investigated whether the allocation behavior of minority and majority group members differed depending on the social category of the receiver. Mann–Whitney tests showed that the minority group did not differ in their allocation behavior toward ingroup (*M* = 14.55, *SD* = 2.99, *Mdn* = 15.00) and outgroup (*M* = 14.58, *SD* = 2.56, *Mdn* = 15.00) members, *U* = 4286.50, *Z* = -0.71, *p* = 0.476. The majority group also did not differ in their allocation behavior toward ingroup (*M* = 14.50, *SD* = 2.96, *Mdn* = 15.00) and outgroup (*M* = 14.32, *SD* = 3.13, *Mdn* = 15.00) members, *U* = 3247.00, *Z* = -0.38, *p* = 0.701.

Using logistic regression, we explored the combined effects of allocator’s group membership, receiver’s group membership, and SVO on tokens allocated to the receiver. Model 1 included the main effects of the three predictors and was significant, *R*^2^ = 14%, *χ*^2^(3) = 31.47, *p* < 0.001. There was a significant main effect of SVO score on allocations, *b* = 0.07, *p* < 0.001, odds ratio = 1.07. This shows that participants who scored high on the SVO measure and were thus more prosocial, were more likely to make a fair allocation to the receiver. Model 2 and Model 3 included the interactions between the predictors and neither was significant, Model 2: *R*^2^ = 15.3%, *χ*^2^(3) = 3.28, *p* = 0.351, and Model 3: *R*^2^ = 15.4%, *χ*^2^(1) = 0.17, *p* = 0.681. There were no significant interactions between the predictors.

Using logistic regression, we also explored the combined effects of allocator’s group membership, receiver’s group membership, and IIM score on tokens allocated to the receiver. Model 1 included the main effects of the three predictors and was not significant, *R*^2^ = 2.7%, *χ*^2^(3) = 5.86, *p* = 0.12. However, there was a significant main effect of IIM score on the tokens allocated to the receiver, *b* = -0.32, *p* = 0.040, odds ratio = 0.73. This shows that participants who identified more with their ingroup were less likely to make a fair allocation to the receiver. Model 2 and Model 3 included the interactions between the predictors and neither was significant, Model 2: *R*^2^ = 4.6%, *χ*^2^(3) = 4.19, *p* = 0.242, and Model 3: *R*^2^ = 4.6%, *χ*^2^(1) = 0.10, *p* = 0.755. There were no significant interactions between the predictors.

#### Social Dominance Orientation (SDO)

A *t*-test was used to compare Chinese and Malay allocators’ SDO scores. As expected, majority group (Malay) allocators (*M* = 44.53, *SD* = 10.40) had significantly higher scores than did minority group (Chinese) allocators (*M* = 41.87, *SD* = 12.85), *t*(367) = -2.17, *p* = 0.031.

Logistic regression was used to explore the combined effects of the three predictors (allocator’s group membership, receiver’s group membership, and SDO score) on allocation behavior. Model 1 included the main effects of conditions and was significant, *R*^2^ = 5%, *χ*^2^(3) = 11.03, *p* = 0.012. Results revealed that there were no effects of allocator’s or receiver’s group membership. The only significant finding was a main effect of SDO, showing that people with a greater preference for group hierarchy were less likely to make fair allocations, *b* = -0.04, *p* = 0.003, odds ratio = 0.96. Model 2 and Model 3 included the interactions between the predictors and neither was significant, Model 2: *R*^2^ = 5.2%, *χ*^2^(3) = 0.47, *p* = 0.926 and Model 3: *R*^2^ = 5.3%, *χ*^2^(1) = 0.17, *p* = 0.683. There were no significant interactions between the predictors.

#### Anticipated Emotions as a Mediator

**FIGURE 3:**
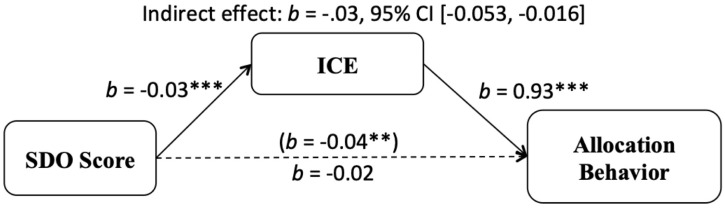
Indirect effect of social dominance orientation on dichotomized averaged tokens allocated to the receiver (irrespective of ethnicity) in the Ultimatum Game through ICE. **p < 0.01, ***p < 0.001 (Study 2).

Using the PROCESS macro (Model 4), we examined whether this association between allocator’s SDO and allocation behavior was mediated by the ICE measure of anticipated emotions (see Figure 3). Consistent with the results already reported, this showed that the overall association between SDO and tokens allocated in UG was negative and significant. There was also a significant negative association between SDO and ICE, showing that participants scoring higher on SDO anticipated fewer cooperative emotions. Furthermore, there was a positive and significant effect of ICE on allocation behavior. Importantly, the indirect association between SDO and allocation behavior through ICE was significant, *b* = -0.03, 95% CI [-0.053, -0.016], and the overall association between SDO and allocation was no longer significant when controlling for SDO, suggesting full mediation.

Finally, using the PROCESS macro (Model 4), we again examined whether the effect of allocator’s SVO on allocation behavior was mediated by anticipated emotions. The mediation analysis showed that the effect of SVO on tokens allocated in UG was significant and positive, *b* = 0.07, 95% CI [0.043, 0.092]. SVO was a significant predictor of ICE, *b* = 0.03, 95% CI [0.022, 0.044], and ICE was a significant predictor of allocations, *b* = 0.84, 95% CI [0.464, 1.224]. In addition, the indirect effect of SVO on allocation behavior through ICE was significant, *b* = 0.03, 95% CI [0.014, 0.049]. However, the direct effect of SVO on allocation remained significant, *b* = 0.05, 95% CI [0.028, 0.079], suggesting partial rather than full mediation.

### Discussion

Contrary to predictions, there was no significant difference in participants’ allocation behavior toward ingroup and outgroup members in Study 2. This was despite the fact that we changed from a within-subjects design in Study 1 to a between-subjects design in Study 2, with a view to eliminating (or at least reducing) social desirability effects. Furthermore, Study 2 had a larger sample in an effort to increase statistical power. The current findings therefore failed to replicate previous research in which ingroup favoritism was found in social dilemmas assessing cooperation (Ben-Ner et al., 2009; Balliet et al., 2014). This may reflect something about the specific cultural context in which the studies were conducted, a point that we will return to below.

We also sought to replicate the difference in allocation behavior between majority and minority allocators found in Study 1, and explored whether SDO played a role as a moderator of the relationship between majority or minority group membership and allocation behavior. However, the findings of Study 2 showed no differences between majority and minority allocation behavior toward others (regardless of ethnicity).

Nevertheless, majority group participants did score higher on SDO than minority group participants, as predicted. Furthermore, there was a significant relation between SDO scores and allocation behavior, such that those higher in SDO were less likely to be fair in allocating tokens to others. This shows that SDO shapes allocation behavior, and further analyses showed that this effect of SDO on allocation behavior toward others was fully mediated by anticipated emotions. This suggests that those who prefer a hierarchical social order are willing to distribute resources unequally because they do not anticipate feeling negative emotions about doing so.

A possible explanation for the fact that there was no difference in allocation behavior between majority and minority group members is the use of the UG instead of the DG in Study 2. The UG is known to have strategic component, in the sense that allocators face a risk of their proposed allocation of resources being rejected by the receiver (Charness and Gneezy, 2008). Although this strategic component typically encourages allocators to offer more than the minimal amount to receivers in order to avoid rejection (Scheres and Sanfey, 2006), there was no evidence that participants were more generous in their allocations in Study 2 than they were in Study 1, which casts doubt on this explanation.

Another possible explanation for the absence of the predicted effects of social category and majority–minority group status is that all participants were university students. Although students are segregated based on religion and hold stereotypes about outgroup members (Mustapha et al., 2009; Tey et al., 2009), it is also the case that students are more tolerant about multi-ethnic interactions than are secondary school students (Tey et al., 2009). The latter researchers found that University of Malaya students’ perceptions of inter-ethnic relations became more positive between 2002 and 2008, although there was still evidence of ethnocentrism among these students (Tey et al., 2009). In addition, researchers found that university students do not see ethnic tension as a racial issue, but rather they believe it has become a norm in the Malaysian society (Mustapha et al., 2009). The same researchers argued that because of their academic background university students are more tolerant and understanding toward other ethnic groups (Mustapha et al., 2009).

## General Discussion

The main aim of these studies was to systematically vary the ethnic group membership of receivers in an economic game setting to see whether this would influence participants’ allocation behavior. There was no evidence of this predicted effect in either study. As noted above, the absence of ingroup bias in allocation behavior may have been due to impression management and social desirability concerns (Study 1) or the use of a university student sample that is likely to have more liberal social attitudes (Study 2). A further possibility is that the manipulation of group membership (through the use of ethnically marked names) was too subtle, although the strong link between ethnicity and the names used makes this less plausible. Given the consistent lack of any empirical support for the predicted effect of group membership, another possibility is that the influence of group membership on allocation behavior in economic games is absent in the Malaysian context, despite the fact that it has been found in other cultural contexts (Whitt and Wilson, 2007; Efferson et al., 2008). A final possibility is that by individualizing the receiver (by giving him or her a name), the procedure used in the current studies may have inadvertently enhanced fair behavior, because participants may have been more reluctant to act unfairly toward a named individual than they would have been if the recipient had been anonymous (as recipients generally are in economic games). Past research has indeed found that DG allocators give more to a named receiver than to an anonymous one (Charness and Gneezy, 2008), although research has also shown that the effect of identifiability on allocators’ generosity in intergroup DGs varies as a function of intergroup relations, such that the tendency to be more generous to identifiable ingroup members is not found in cohesive groups, perhaps because in highly cohesive groups, the prototypicality of a group member is more important than his or her personal attributes (Ritov and Kogut, 2017).

A second objective of Study 2 was to investigate possible differences in allocations made by members of majority and minority groups. Interestingly, the results of Study 1 appeared to show that allocators belonging to minority groups were more generous toward others (regardless of the receiver’s ethnicity) than were majority group members, perhaps reflecting a stronger preference for equality in social relations (Pratto et al., 1994). However, this pattern of findings was not replicated in Study 2. However, Study 2 did reveal that majority group members had higher SDO scores, and SDO was negatively related to allocation behavior. Thus, the current studies provide suggestive evidence that belonging to a majority or minority social group may play a role in resource allocation behavior, through the relation between group status and SDO, and the effect of SDO on allocation behavior. However, it is of course true that the Malays differ from the Chinese and Indians with regard to many other aspects besides their majority versus minority group membership (for example, they differ in cultural norms). Thus, future research should include a broader range of majority and minority groups.

Furthermore, the effect of group membership status on allocation behavior in Study 1 and the relation between SDO and allocation behavior in Study 2 were both fully mediated by anticipated emotions. As noted above, full mediation suggests that the influence of individual differences in preferences for a hierarchical social order on individual and institutional discrimination operates through their effect on cooperative and competitive emotions. This provides additional support for the general argument that anticipated emotions play a key role in the link between dispositional preferences and resource allocation behavior.

Most studies in the psychological literature study samples from populations that can be characterized as WEIRD (Western, Educated, Industrialized, Rich, and Democratic), meaning that the findings may not generalize to people living in the rest of the world (Henrich et al., 2010). One of the strengths of the current research is that we replicated some of the key findings reported by Bono et al. (unpublished) using a population from a non-Western country. This helps to establish the generalizability of the mediating role played by anticipated emotions in the relation between SVO and allocation behavior.

Some limitations of the current studies need to be acknowledged. The first names used to manipulate receiver ethnicity were not pre-tested with respect to their perceived cooperativeness or friendliness, which could conceivably influence allocation decisions (Nett et al., 2020). Future studies using a similar procedure could have the names rated on these dimensions to rule out this possibility. Another possible limitation is that allocators and receivers were always of the same gender. It may well be that there are differences in mixed-sex versus same-sex dyads (Eagly and Crowley, 1986). Against this limitation, it could be argued that keeping the same-gender interaction consistent is a strength of our procedure because it avoids introducing another layer of social categorization.

A limitation of Study 2 is the fact that it did not investigate whether participants differed in their anticipated cooperative and competitive emotions in relation to ingroup and outgroup members. This was because the ICE measure used in Study 2 was not customized to the ethnic identity of the receivers. Dropping the manipulation of receiver’s social identity in the ICE measure used in Study 2 was driven by the need to reduce the length of time needed to complete the study. Future studies could seek to examine the extent to which respondents anticipate cooperative and competitive emotions when allocating resources to an ingroup or outgroup member. This might provide further insight into the role of anticipated emotions in mediating the effect of SDO on allocation behavior.

## Conclusion

Although the central manipulation of receiver’s social identity did not influence allocation behavior in the expected way, there was some indication that the majority/minority status of allocators and their individual difference in preference for status hierarchies does influence allocations decisions. This reveals that these factors need to be taken into account in research on allocation behavior in multicultural settings. Also, the fact that anticipated emotions played a significant role in mediating the relation between SVO and allocation behavior is consistent with the notion that anticipated emotions play a key role in resource allocation decision making. Finally, the finding that the negative effect that SDO had on allocations toward others was also mediated by anticipated emotions speaks to the more general role of anticipated emotions as a psychological mechanism that can explain how prosocial and proself dispositions are expressed in behavior.

## Data Availability Statement

The datasets generated for these studies are available at https://osf.io/fqx2p/?view_only=cbf73bad3a634aff802ce22f7761a566.

## Ethics Statement

The studies involving human participants were reviewed and approved by Cardiff University’s School of Psychology Ethics Committee. The patients/participants provided their written informed consent to participate in this study.

## Author Contributions

All authors listed, have made a substantial, direct, and intellectual contribution to the work, and approved it for publication. SB designed the experiments, collected and analyzed the data, and wrote the manuscript. JS and AM provided input for the design of the experiments, assisted with the analyses of data and gave feedback on the writing of the manuscript.

## Conflict of Interest

The authors declare that the research was conducted in the absence of any commercial or financial relationships that could be construed as a potential conflict of interest.

## References

[B1] Balliet D. Parks C. Joireman J. (2009). Social value orientation and cooperation in social dilemmas: a meta-analysis. *Group Process. Intergroup Relat.* 12 533–547. 10.1177/1368430209105040

[B2] Balliet D. Wu J. De Dreu C. K. (2014). Ingroup favoritism in cooperation: a meta-analysis. *Psychol. Bull.* 140 1556–1581. 10.1037/a0037737 25222635

[B3] Ben-Ner A. McCall B. P. Stephane M. Wang H. (2009). Identity and in-group/out-group differentiation in work and giving behaviors: experimental evidence. *J. Econ. Behav. Organ.* 72 153–170. 10.1016/j.jebo.2009.05.007

[B4] Berg J. Dickhaut J. McCabe K. (1995). Trust, reciprocity, and social history. *Games Econ. Behav.* 10 122–142. 10.1006/game.1995.1027

[B5] Bono S. A. (2018). *Social Value Orientation and Anticipated Emotions in Resource Allocation Decisions.* PhD dissertation, Cardiff University, Cardiff. 10.3389/fpsyg.2020.01455 PMC7358597 32733327

[B6] Bono S. A. Van der Schalk J. Manstead A. (2019). The effects of intergroup relations on anticipated emotions and resource allocation in a multi-ethnic Malaysian sample. *Paper presented at the 3rd International Conference of Social Sciences USM*, (Malaysia: Penang).

[B7] Brown G. K. (2007). Making ethnic citizens: the politics and practice of education in Malaysia. *Int. J. Educ. Dev.* 27 318–330. 10.1016/j.ijedudev.2006.12.002

[B8] Charness G. Gneezy U. (2008). What’s in a name? Anonymity and social distance in dictator and ultimatum games. *J. Econ. Behav. Organ.* 68 29–35. 10.1016/j.jebo.2008.03.001

[B9] Cheong K.-C. Hill C. Leong Y.-C. (2016). Malaysia’s education policies and the law of unintended consequences. *J. Int. Comp. Educ.* 5 73–85. 10.14425/jice.2016.5.2.73

[B10] Cohen J. (1969). *Statistical Power Analysis for the Behavioural Sciences.* New York, NY: Academic Press.

[B11] De Hooge I. E. Breugelmans S. M. Zeelenberg M. (2008). Not so ugly after all: when shame acts as a commitment device. *J. Pers. Soc. Psychol.* 95 933–943. 10.1037/a0011991 18808269

[B12] Department of Statistics (2018). *Current Population Estimates Malaysia 2017-2018.* Available online at: https://www.dosm.gov.my/v1/index. php?r=column/cthemeByCat&cat=155&bul_id=c1pqTnFjb29HSnNYNUpiTm NWZHArdz09&menu_id=L0pheU43NWJwRWVSZklWdzQ4TlhUUT09 (accessed January 15, 2018).

[B13] Dorfman A. Eyal T. Bereby-Meyer Y. (2014). Proud to cooperate: the consideration of pride promotes cooperation in a social dilemma. *J. Exp. Soc. Psychol.* 55 105–109. 10.1016/j.jesp.2014.06.003

[B14] Eagly A. H. Crowley M. (1986). Gender and helping behavior: a meta-analytic review of the social psychological literature. *Psychol. Bull.* 100 283–308. 10.1037/0033-2909.100.3.283 38376350

[B15] Efferson C. Lalive R. Fehr E. (2008). The coevolution of cultural groups and ingroup favoritism. *Science* 321 1844–1849. 10.1126/science.1155805 18818361

[B16] Faul F. Erdfelder E. Buchner A. Lang A.-G. (2009). Statistical power analyses using G^∗^ Power 3.1: tests for correlation and regression analyses. *Behav. Res. Methods* 41 1149–1160. 10.3758/brm.41.4.1149 19897823

[B17] Faul F. Erdfelder E. Lang A.-G. Buchner A. (2007). G^∗^ Power 3: a flexible statistical power analysis program for the social, behavioral, and biomedical sciences. *Behav. Res. Methods* 39 175–191. 10.3758/BRM.41.4.1149 17695343

[B18] Fiedler S. Hellmann D. M. Dorrough A. R. Glöckner A. (2018). Cross-national in-group favoritism in prosocial behavior: evidence from Latin and North America. *Judgm. Decis. Mak.* 13, 42–60.

[B19] Forsythe R. Horowitz J. L. Savin N. E. Sefton M. (1994). Fairness in simple bargaining experiments. *Games Econ. Behav.* 6 347–369. 10.1006/game.1994.1021

[B20] Gill D. Prowse V. (2012). A structural analysis of disappointment aversion in a real effort competition. *Am. Econ. Rev.* 102 469–503. 10.1257/aer.102.1.469

[B21] Guimond S. Dambrun M. Michinov N. Duarte S. (2003). Does social dominance generate prejudice? Integrating individual and contextual determinants of intergroup cognitions. *J. Pers. Soc. Psychol.* 84 697–721. 10.1037/0022-3514.84.4.697 12703644

[B22] Güth W. Schmittberger R. Schwarze B. (1982). An experimental analysis of ultimatum bargaining. *J. Econ. Behav. Organ.* 3 367–388. 10.1016/0167-2681(82)90011-7

[B23] Haesevoets T. Folmer C. R. Van Hiel A. (2015). Cooperation in mixed-motive games: the role of individual differences in selfish and social orientation. *Eur. J. Pers.* 29 445–458. 10.1002/per.1992

[B24] Haidt J. (2003). The moral emotions. *Handb. Affect. Sci.* 11 852–870.

[B25] Henrich J. Heine S. J. Norenzayan A. (2010). Most people are not WEIRD. *Nature* 466:29. 10.1038/466029a 20595995

[B26] Hinkle S. Taylor L. A. Fox-Cardamone D. L. Crook K. F. (1989). Intragroup identification and intergroup differentiation: a multicomponent approach. *Br. J. Soc. Psychol.* 28 305–317. 10.1111/j.2044-8309.1989.tb00874.x

[B27] Ho A. K. Sidanius J. Kteily N. Sheehy-Skeffington J. Pratto F. Henkel K. E. (2015). The nature of social dominance orientation: theorizing and measuring preferences for intergroup inequality using the new SDO7 scale. *J. Pers. Soc. Psychol.* 109 1003–10028. 26479362 10.1037/pspi0000033

[B28] Jomo K. S. Sundaram J. K. (2004). *The New Economic Policy and Interethnic Relations in Malaysia.* Geneva: UNRISD.

[B29] Kahneman D. Knetsch J. L. Thaler R. H. (1986). Fairness and the assumptions of economics. *J. Bus.* 59 S285–S300.

[B30] Ketelaar T. Tung Au W. (2003). The effects of feelings of guilt on the behaviour of uncooperative individuals in repeated social bargaining games: an affect-as-information interpretation of the role of emotion in social interaction. *Cogn. Emot.* 17 429–453. 10.1080/02699930143000662 29715746

[B31] Khattab U. M. (2016). Who are the diasporas in Malaysia? The discourse of ethnicity and Malay(sian) identity. *Sosiohumanika* 3 157–174.

[B32] Leach C. W. Van Zomeren M. Zebel S. Vliek M. L. Pennekamp S. F. Doosje B. (2008). Group-level self-definition and self-investment: a hierarchical (multicomponent) model of in-group identification. *J. Pers. Soc. Psychol.* 95 144–165. 10.1037/0022-3514.95.1.144 18605857

[B33] Levin S. Sidanius J. (1999). Social dominance and social identity in the United States and Israel: ingroup favoritism or outgroup derogation? *Polit. Psychol.* 20 99–126. 10.1111/0162-895x.00138

[B34] Liebe U. Tutic A. (2010). Status groups and altruistic behaviour in dictator games. *Rational. Soc.* 22 353–380. 10.1177/1043463110366232

[B35] Mellers B. A. McGraw A. P. (2001). Anticipated emotions as guides to choice. *Curr. Direct. Psychol. Sci.* 10 210–214. 10.1111/1467-8721.00151

[B36] Messick D. M. McClintock C. G. (1968). Motivational bases of choice in experimental games. *J. Exp. Soc. Psychol.* 4 1–25. 10.1016/0022-1031(68)90046-2

[B37] Mokhtar K. S. Chan A. R. Singh P. S. J. (2017). The new economic policy (1970–1990) in Malaysia: the economic and political perspectives. *Int. J. Media Commun.* 1 12–17. 10.5176/2251-2403_PSSIR12.50

[B38] Montesino M. U. (2012). Cross-cultural conflict and affirmative action: inter-and intra-ethnic dilemmas of Malaysia’s heterogeneous workplace. *Int. J. Cross Cult. Manag.* 12 115–132. 10.1177/1470595811413110

[B39] Murphy R. O. Ackermann K. A. Handgraaf M. (2011). Measuring social value orientation. *Judg. Decis. Making* 6 771–781. 10.2139/ssrn.1804189

[B40] Mustapha R. Azman N. Karim F. Ahmad A. R. Lubis M. A. (2009). Social integration among multi-ethnic students at selected Malaysian universities in Peninsular Malaysia: a survey of campus social climate. *Asean J. Teach. Learn. High. Educ.* 1 35–44.

[B41] Nett T. Dorrough A. Jekel M. Glöckner A. (2020). Perceived biological and social characteristics of a representative set of German first names. *Soc. Psychol.* 51 1–17.

[B42] Niedenthal P. M. Tangney J. P. Gavanski I. (1994). “If only I weren’t” versus “If only I hadn’t”: distinguishing shame and guilt in counterfactual thinking. *J. Pers. Soc. Psychol.* 67 585–595. 10.1037/0022-3514.67.4.585 7965606

[B43] Pietsch J. Clark M. (2014). Citizenship rights in Malaysia: the experience of social and institutional discrimination among ethnic minorities. *Citizenship Stud.* 18 303–314. 10.1080/13621025.2014.905270

[B44] Pletzer J. L. Balliet D. Joireman J. Kuhlman D. M. Voelpel S. C. Van Lange P. A. M. (2018). Social value orientation, expectations, and cooperation in social dilemmas: a meta-analysis. *Eur. J. Pers.* 32 62–83. 10.1002/per.2139

[B45] Pratto F. Sidanius J. Stallworth L. M. Malle B. F. (1994). Social dominance orientation: a personality variable predicting social and political attitudes. *J. Pers. Soc. Psychol.* 67 741–763. 10.1037/0022-3514.67.4.741

[B46] Ritov I. Kogut T. (2017). Altruistic behavior in cohesive social groups: the role of target identifiability. *PLoS One* 12:e0187903. 10.1371/journal.pone.0187903 29161282 PMC5697805

[B47] Scheres A. Sanfey A. G. (2006). Individual differences in decision making: drive and reward responsiveness affect strategic bargaining in economic games. *Behav. Brain Funct.* 2:35. 10.1186/1744-9081-2-35 17049091 PMC1635418

[B48] Sidanius J. Pratto F. Mitchell M. (1994). In-group identification, social dominance orientation, and differential intergroup social allocation. *J. Soc. Psychol.* 134 151–167. 10.1080/00224545.1994.9711378

[B49] Siddique S. Suryadinata L. (1981). Bumiputra and pribumi: economic nationalism (indiginism) in Malaysia and Indonesia. *Pac. Affairs* 54 662–687. 10.2307/2757890

[B50] Tajfel H. Turner J. (1979). “An integrative theory of intergroup conflict,” in *The Social Psychology of Intergroup Relations*, eds Austin W. G. Worehel S. (Montery: Brooks/Cole), 33–47.

[B51] Tey N.-P. Awang H. Singaravelloo K. (2009). Ethnic interactions among students at the University of Malaya. *Malaysian J. Econ. Stud.* 46 53–74.

[B52] Tracy J. L. Robins R. W. (2007). The psychological structure of pride: a tale of two facets. *J. Pers. Soc. Psychol.* 92 506–525. 10.1037/0022-3514.92.3.506 17352606

[B53] Tyson A. D. Jeram D. Sivapragasam V. Azlan H. N. (2017). Ethnicity, education and the economics of brain drain in Malaysia: youth perspectives. *Malaysian J. Econ. Stud.* 48 175–184.

[B54] Van Der Schalk J. Bruder M. Manstead A. (2012). Regulating emotion in the context of interpersonal decisions: the role of anticipated pride and regret. *Front. Psychol.* 3:513. 10.3389/fpsyg.2012.00513 23293615 PMC3536270

[B55] Van der Schalk J. Kuppens T. Bruder M. Manstead A. S. R. (2015). The social power of regret: the effect of social appraisal and anticipated emotions on fair and unfair allocations in resource dilemmas. *J. Exper. Psycho. Gen.* 144:151. 10.1037/xge0000036 25384163 PMC4312135

[B56] Van Lange P. A. De Bruin E. Otten W. Joireman J. A. (1997). Development of prosocial, individualistic, and competitive orientations: theory and preliminary evidence. *J. Pers. Soc. Psychol.* 73 733–746. 10.1037/0022-3514.73.4.733 9325591

[B57] Van Osch Y. Zeelenberg M. Breugelmans S. M. (2018). The self and others in the experience of pride. *Cogn. Emot.* 32 404–413. 10.1080/02699931.2017.1290586 28278739

[B58] Whitt S. Wilson R. K. (2007). The dictator game, fairness and ethnicity in postwar Bosnia. *Am. J. Polit. Sci.* 51 655–668. 10.1111/j.1540-5907.2007.00273.x

[B59] Wietzker A. Buysse A. Loeys T. Brondeel R. (2012). Easing the conscience: feeling guilty makes people cooperate in divorce negotiations. *J. Soc. Pers. Relationsh.* 29 324–336. 10.1177/0265407511431180

[B60] Zeelenberg M. Pieters R. (2007). A theory of regret regulation 1.0. *J. Consumer Psychol.* 17 3–18. 10.1207/s15327663jcp1701_3

[B61] Zeelenberg M. Van Dijk W. W. Manstead A. S. R. Van Der Pligt J. (2000). On bad decisions and disconfirmed expectancies: the psychology of regret and disappointment. *Cogn. Emot.* 14 521–541. 10.1080/026999300402781

